# Incongruous Harmonics of Vibrating Solid‐Solid Interface

**DOI:** 10.1002/smll.202409410

**Published:** 2024-11-17

**Authors:** Pardis Biglarbeigi, Alessio Morelli, Gourav Bhattacharya, Joanna Ward, Dewar Finlay, Nikhil Bhalla, Amir Farokh Payam

**Affiliations:** ^1^ Department of Pharmacology & Therapeutics University of Liverpool Whelan Building, Liverpool England L69 3GE UK; ^2^ Nanotechnology and Integrated Bioengineering Centre School of Engineering Ulster University Belfast BT15 1AP UK

## Abstract

Deconvoluting the vibrations and harmonics in solid‐solid interfaces is crucial for designing materials with improved performance, durability, and functionality. The measured vibrating microcantilever signal in the dynamic atomic force microscopy (AFM) encompasses a multitude of distinct signatures reflecting a diverse array of material properties. Nevertheless, uncertainties persist in decoding these signatures, primarily arising from the interplay between attractive and repulsive forces. Consequently, it is challenging to correlate the generated harmonics within the solid‐solid interfaces with the imaged phase and topography of materials, as well as the occasional observed contrast reversal. In this study, the vibration harmonics produced at solid‐solid interfaces are correlated, linking them to short‐range nano‐mechanical characteristics through a comprehensive blend of theory, simulation, and experimental methods. These findings shed light on the roots of harmonic generation and contrast reversals, opening avenues for designing innovative materials with customized properties.

## Graphical abstract

This study presents an integrated simulations and experiments methodology to analyze harmonic responses in vibrating microcantilevers at solid interfaces, revealing the roles of attractive and repulsive forces as well as interaction regime transitions. This approach links harmonics to nano‐mechanical properties, aiding material design with enhanced performance, durability, and tailored characteristics.

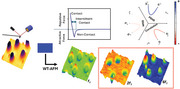

## 1 Introduction

Surface interactions and material properties involved in vibrating solid‐solid interfaces, play a key role across disciplinary boundaries, serving as a unifying theme that underpins advancements in physics, chemistry, biomechanics, and materials science.^[^ ^1^, ^2^, ^3^, ^4^, ^5^ ^]^ In physics and mechanics, understanding the surface interactions of materials is crucial for understanding phenomena such as adsorption, stiffness, and electronic properties.^[^ ^6^, ^7^, ^8^ ^]^ The behavior of materials at the nanoscale is heavily influenced by surface forces, impacting the design and functionality of advanced technologies.^[^ ^9^, ^10^ ^]^ In chemistry, surface interactions are integral to elucidating reaction mechanisms and the development of new materials with tailored properties.^[^ ^11^, ^12^, ^13^, ^14^ ^]^ The exploration of material properties at the atomic and molecular levels is central to advancing our comprehension of chemical processes. Elsewhere in the field of biomechanics, the interplay between biological tissues and external surfaces is paramount for comprehending physiological functions, such as cell adhesion, tissue regeneration, and biomaterial compatibility.^[^ ^15^, ^16^, ^17^, ^18^ ^]^ Additionally, the mechanical properties of surfaces and materials at solid‐solid interface influence medical device design and the development of innovative strategies for tissue engineering.^[^ ^15^, ^19^ ^]^

Since its invention, atomic force microscopy (AFM) has become the mainstream analytical tool capable of imaging, characterization, and manipulation of materials at the nanoscale. Recent advancements in AFM technology makes AFM a de‐facto instrument to study surface interactions and material properties.^[^ ^20^ ^]^ Due to its easy use, preserving tip and sample from damage, ability to use as an imaging and spectroscopy tool, and dynamics nature, amplitude modulation AFM (AM‐AFM), also known as tapping mode AFM, is the most commonly used AFM technique. Furthermore, combining AM‐AFM with other modes and methods (e.g., multifrequency AFM, kelvin probe force microscopy (KPFM), etc), leads to simultaneously mapping and quantifying Young's modulus, Hamaker constant, electrostatic charges, or surface potential differences.^[^ ^21^, ^22^, ^23^, ^24^ ^]^ Although significant research exists on AFM modalities and applications, as well as its nonlinear behavior; theoretical contribution devoted to the harmonics’ generation and the relationship of material properties, dynamics of microcantilever and harmonics arise from the interaction between the vibrating cantilever‐tip ensemble and surface of materials are still incomplete. In dynamic AFM, the measured data are captured from the excitation and detection of a single frequency of cantilever signal, mainly the fundamental frequency of cantilever. Whereas in multifrequency AFM the data are collected from the different frequencies like eigenmodes that conventionally are detected using lock‐in amplifiers (LIAs) and phase lock loops (PLL).^[^ ^22^ ^]^ However, the information of the sample properties included in the other frequency components, like harmonics, is irreversibly lost that limits the capabilities of the AFM.^[^ ^25^, ^26^, ^27^ ^]^ In fact, high harmonics have long been recognized, but its utilisation has been extremely limited due to theoretical and experimental complexity.^[^ ^28^, ^29^, ^30^, ^31^, ^32^, ^33^, ^34^ ^]^ Thanks to recently developed wavelet transform‐based AFM (WT‐AFM),^[^ ^21^, ^35^ ^]^ the experimental complexity to extract frequency components of cantilever signal is compensated and now simultaneous extraction of all frequency components of the measured cantilever signal is possible. This advancement provides a significant opportunity to study the nature of higher harmonics of cantilever interacting with materials surfaces.

Considering the recent progress, regardless of utilizing harmonics to extract material properties, there are still challenges regarding the fundamentals of harmonics in AFM such as the interplay between harmonics and material properties, AFM feedback system, and image contrast. As an example, measurements show reverse contrast between second harmonic and other harmonics’ images especially in air environment.^[^ ^31^, ^33^, ^36^ ^]^ Moreover, most of the harmonic's studies on AFM focus on repulsive mode and materials’ stiffness, especially in liquid environments,^[^ ^37^, ^38^, ^39^, ^40^, ^41^, ^42^, ^43^ ^]^ while the effect of different material properties and both attractive/repulsive interaction regimes on the harmonic's response are not completely understood. To address these problems, in this work using integration of our developed WT‐based AFM with simulation and data analysis approaches, based on digital twin concept, we study the interplay between viscosity, Young's modulus, Hamaker constant, and capillary force on the harmonic response of cantilever and explain the contrast obtained from different materials on the harmonics’ images of AFM.

## 2 Results and Discussion

**Figure 1:**
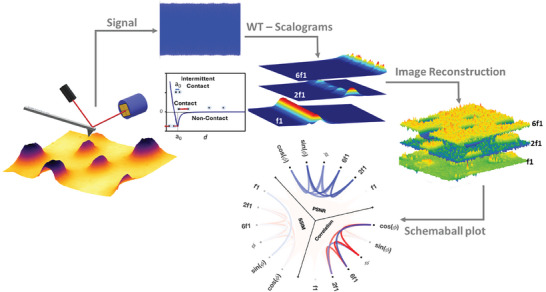
Incongruous AFM harmonics Schematic of the proposed framework to analyze the interplay between interaction forces, materials properties, and the harmonic response of dynamics AFM.

The diagram illustrating our WT‐based AFM, designed to capture all frequency components of the measured cantilever signal simultaneously, along with our proposed methodology for examining the interaction between generated harmonics and material properties, is depicted in **Figure** **1**.

Harmonic experiments were conducted using dynamic mode AFM, where the cantilever was excited at its first resonance, and harmonic signals were continuously monitored. Wavelet‐based techniques are commonly employed in signal processing to analyze non‐stationary signals. The Wavelet Transform (WT) is adept at extracting time‐frequency information from time‐domain signals. In this regard, the Continuous Wavelet Transform (CWT) has proven to be a practical tool for analyzing cantilever signals for AFM image reconstruction^[^ ^35^ ^]^ and transient analysis.^[^ ^21^ ^]^

In this study, the CWT is utilized to extract the harmonic responses of the cantilever's signal and facilitate image reconstruction, enabling the simultaneous analysis of material properties by examining the frequency components of the cantilever signal. However, owing to its limitations in filter bank design, CWT may not offer high‐quality information regarding higher frequency components of the signal. The Maximal Overlap Discrete Wavelet Packet Transform (MODWPT)^[^ ^44^ ^]^ offers a solution by decomposing the signal into frequency pass‐bands. This decomposition ensures that the calculated coefficients at each frequency band align precisely with the original signal, thanks to its zero‐phase filtering. Consequently, the associated frequency band coefficients are analyzed through CWT scalograms to obtain the amplitudes of higher frequency components of the signal. Additionally, images for the amplitude and phase at the cantilever excitation frequency, as well as the amplitudes of the second (2*f*_1_) and sixth harmonics (6*f*_1_), are computed.

The reconstructed images for all frequency components are further considered to analyze the interplay between interaction forces and material properties by calculating three objective image quality metrics, i.e., correlation, peak signal‐to‐noise ratio (PSNR), and structural similarity index measure (SSIM). Further information is given in materials and methods. We also performed cascaded Principal Component Analysis (cPCA) to find relationship between the material physical properties and the AFM signal measurands. To comprehensively examine the dynamic behavior of AFM and its harmonic response in relation to material properties, and to offer comparison and theoretical insights for our experimental observations, we conducted simulation‐informed experiments.

For simulations, we model the cantilever‐tip motion in dynamic AFM, representing the signal from the photodetector, as a driven and damped point‐mass oscillator subjected to both conservative and non‐conservative forces.^[^ ^45^ ^]^ To obtain numerical solutions, we employ a fourth‐order Runge–Kutta algorithm implemented in C++ software. More comprehensive information is provided in the materials and methods section. Since the second and sixth harmonics exhibit distinct behaviors as the main detected amplitudes, our focus was primarily on studying them. Our observations, detailed in the Supporting Information, suggest that our findings regarding these harmonics can be extrapolated to other harmonics as well. In our experiments, we employed four distinct samples, consisting of six different materials with diverse properties. The first sample, Polystyrene‐Low Density Polyethylene (PS‐LDPE), exhibits varying Young's modulus and viscosity: LDPE (0.11 ± 0.02 GPa and 37–40 Pas)^[^ ^46^ ^]^ and PS (2.1 ± 0.1 Gpa, 418 ± 100 Pas)^[^ ^46^ ^]^, while maintaining similar Hamaker constants (50–100 zJ).^[^ ^45^ ^]^ The second sample, Polystyrene‐ Poly (methyl methacrylate) (PS‐PMMA), shares similar Young's modulus and Hamaker constant (2–3 GPa and 50–100 zJ)^[^ ^45^, ^46^, ^47^, ^48^ ^]^ but differs in viscosity (PS: 418 ± 100 Pas, PMMA: 186 ± 81 Pas)).^[^ ^46^ ^]^ The third sample comprises nano gold deposited on Highly ordered pyrolytic graphite (HOPG), featuring distinct Young's modulus while both materials are considered hard (Au: 75 ± 4 GPa, HOPG: 20 ± 3 GPa)^[^ ^47^, ^48^ ^]^ and different Hamaker constants (Au: 250 ± 50 zJ, HOPG: 150 ± 50 zJ).^[^ ^48^, ^49^ ^]^ The fourth sample, Graphene Oxide (GO) deposited on HOPG, exhibited different high Young's modulus (GO: 207.6 ± 23.4 GPa, HOPG: 20 ± 3 GPa) and Hamaker constant (GO: 50 ± 20 zJ, HOPG: 150 ± 50 zJ).^[^ ^9^, ^50^ ^]^ We conducted measurements on PS‐LDPE and PS‐PMMA at various free and setpoint amplitudes. As observed in previous reports,^[^ ^29^, ^38^, ^51^ ^]^ we anticipate a consistent trend where higher free amplitudes and lower setpoints result in an increase in harmonics (see Supporting Information).

**Figure 2:**
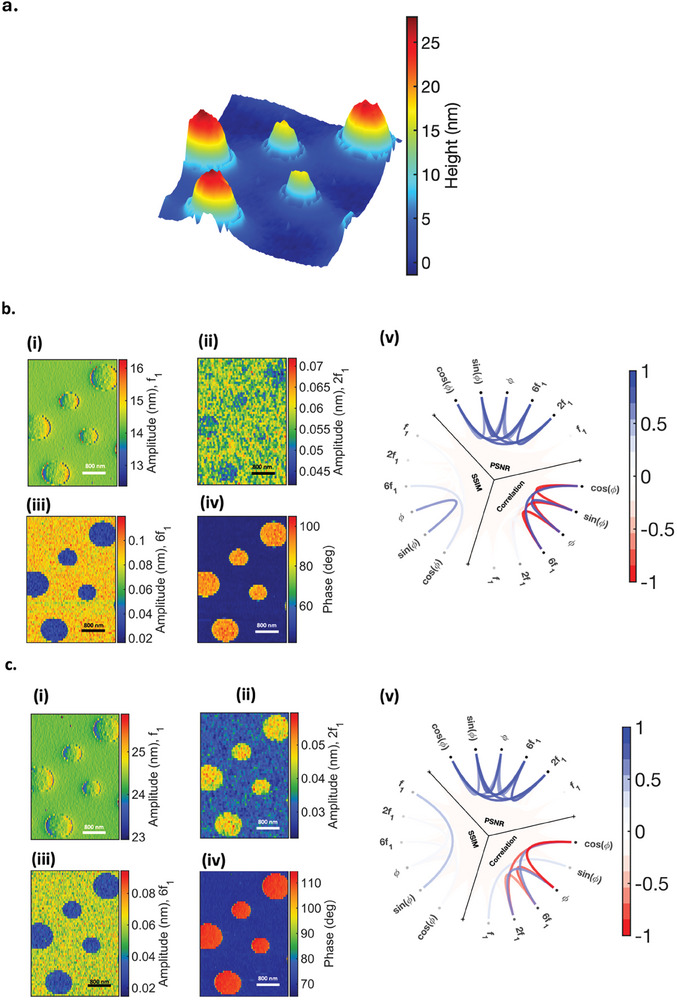
PS‐LDPE sample. a). topography scanned over 4µ × 4µ section. b). 31 nm free amplitude for 50% setpoint b.i.‐b.v. showing amplitude, 2nd and 6th harmonics, phase, and schemaball representing the correlation, PSNR, and SSIM between the WT‐derived images. c) 31nm free amplitude for 85% setpoint c.i.‐c.v. showing amplitude, 2nd and 6th harmonics, phase, and schemaball representing the correlation, PSNR, and SSIM between the WT‐derived images.

To analyze the harmonics produced by PS‐LDPE, we present images of topography, amplitude, phase, second, and sixth harmonics in **Figure** **2**, corresponding to a free amplitude of 31 nm and setpoint amplitudes of 85% and 50%. When keeping the free amplitude constant at 31 nm and varying the setpoint (represented in Figure 2b,c), a distinct harmonics response is observed. At lower setpoint amplitudes, both the second and sixth harmonics of LDPE are lower than those of PS, displaying consistent contrast. The normalized PSNR between the second and sixth harmonic shows high values (close to 1), indicating low numerical differences between the two images. The correlation between 2*f*_1 _and 6*f*_1 _, however, shows very small positive values shown in light blue (Figure 2b‐v). Conversely, at higher setpoints (85%), the second harmonic of LDPE is higher than PS, while the sixth harmonic is lower, resulting in a contrast reversal, as visualized in the scheme ball plot as a negative correlation between the second and sixth harmonics (Figure 2c‐v). This trend is consistently observed for various free amplitudes and setpoints chosen for imaging this sample (refer to Supporting Information).

**Figure 3:**
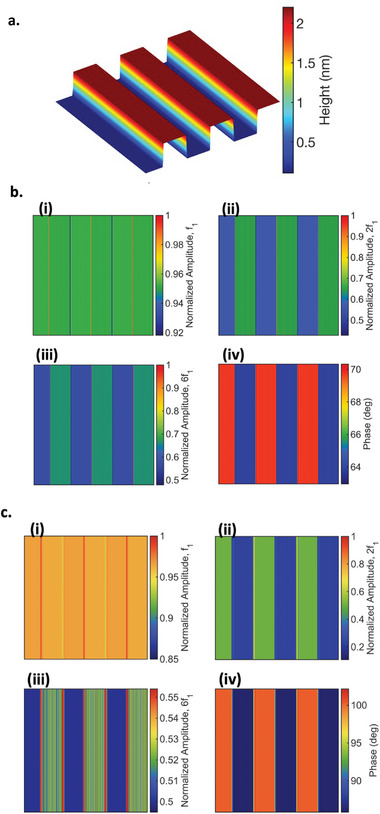
Simulation of PS‐LDPE sample. a) PS‐LDPE surface. b) Fully repulsive interactions i‐iv showing simulated amplitude, 2nd and 6th harmonics, and phase images. c) Simultaneous attractive‐repulsive interactions i‐iv showing simulated amplitude, 2nd and 6th harmonics, and phase images.

Further comparison of the harmonic values for both PS and LDPE reveals interesting findings. In the fully repulsive mode, PS generates higher second harmonics, as expected due to its higher Young's modulus. Conversely, in the attractive/repulsive regime, the second harmonic of LDPE is higher than PS. This can be explained through phase images: for PS, the tip is in the fully repulsive regime, and the harmonics are primarily generated by Young's modulus, while in LDPE, the effect of Hamaker constant, or attractive forces, significantly contributes to the generation of higher second harmonics for LDPE than PS (Figure 2b‐iv). This highlights the significance of attractive forces, mainly van der Waals force, in harmonics generation and consistency between phase and sixth harmonics contrast. Analyzing the harmonics response in correlation with the measured phase image reveals that reducing the setpoint leads to a decrease in phase. This can be attributed to the transition from intermittent contact regime, where both attractive and repulsive forces play a role in the interaction, to a fully repulsive regime, where the repulsive force is the major force in the interaction. It is noteworthy to mention that a lower phase means more repulsive force. Essentially, for these two materials, the cantilever intermittently contacts the surface. Despite indentations, the attractive force significantly contributes to the interaction, leading to an increased contact time between the cantilever and the surface. The high contrast in the phase image is further explained by the substantial difference in viscosity between PS and LDPE, as observed in simulations in **Figure** **3**. Our correlation analysis and simulations (see Figure 6) also indicate that an increase in viscosity leads to a decrease in harmonics, compensating for the effect of the higher Young's modulus of PS, especially in generating a second harmonic.

To verify and validate our hypothesis regarding contrast reversal in PS‐LDPE, we conducted simulations under conditions mirroring those of the experimental setup, with similar properties as PS‐LDPE. The results are presented in Figure 3. In the scenario where the tip is in full repulsive interaction with the PS‐LDPE (Figure 3b), reflected by phase values <90°, we observe a similar behavior to the experiments: the second and sixth harmonics of PS are higher than those of LDPE, as shown in Figure 3b. Conversely, in the case where the tip experiences simultaneous attractive and repulsive interactions (intermittent contact regime), again reflected by phase values exceeding 90°, we observe a similar behavior to the experiments: stiffer materials generate higher sixth harmonics, while softer materials exhibit higher second harmonics, as presented in Figure 3c. These results demonstrate a good sensitivity of second harmonics to attractive forces compared to sixth harmonics and highlight the dominance of Young's modulus in influencing sixth harmonics. It is noteworthy that the sixth harmonic is close to the second eigenfrequency of the cantilever, which suggests the influence of surface stiffness on its excitation and the subsequent contribution of the sixth harmonic to phase shift, as previously discussed.^[^ ^52^, ^53^ ^]^

**Figure 4:**
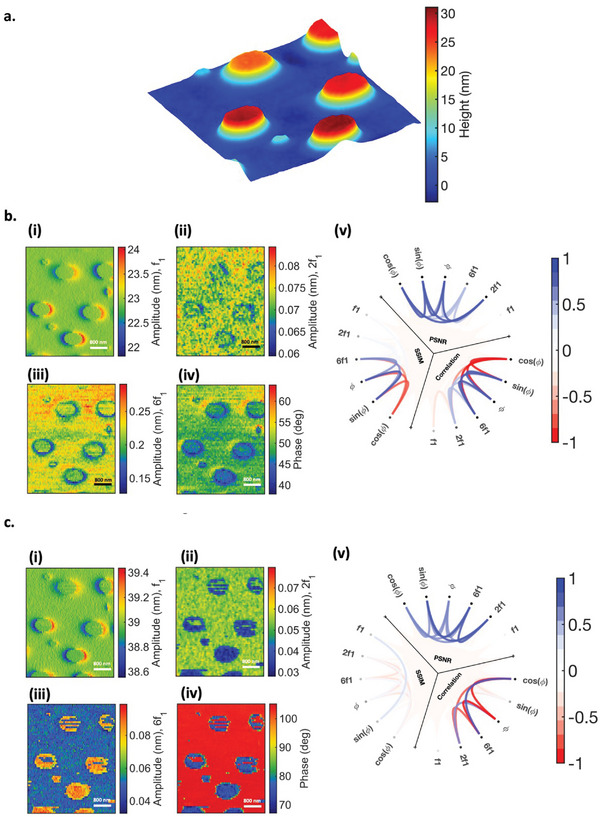
PS‐PMMA sample. a) topography scanned over 2µ × 2µ section, b) 50 nm 50% setting b.i.‐b.v. showing amplitude, 2nd and 6th harmonics, phase, and schemaball representing the correlation, PSNR, and SSIM between the WT‐derived images. c) 50 nm 85% setting c.i.‐c.v. showing amplitude, 2nd and 6th harmonics, phase, and schemaball representing the correlation, PSNR, and SSIM between the WT‐derived images.

The second sample examined in our study was PS‐PMMA (**Figure** **4**). Given the close values of Young's modulus and Hamaker constants for PS and PMMA, we anticipate that the influence of viscosity on changes in phase and harmonics will be evident. In our investigation, we maintained the same free amplitude (at 50 nm) and varied the setpoints (at 50% and 85%). Notably, for the second harmonic, there is a consistent decrease observed for PS compared to PMMA in both cases, as seen in Figure 3b‐ii,c‐ii. This decrease can be attributed to the slightly higher Young's modulus of PMMA and the nearly identical Hamaker constants of both samples. However, a notable discrepancy is observed for the sixth harmonic.

At lower setpoints (50%), where the phase image indicates that for PMMA, the repulsive regime predominates over PS, the sixth harmonic of PS is lower than that of PMMA (Figure 3b‐iii). In both samples, the cantilever operates in repulsive contact, as indicated by the phase image as well. In contrast, for cases where there is a contrast reversal between the second and sixth harmonics, represented by negative correlation as seen in Figure 4c‐v, PS is in a repulsive regime while PMMA is in an intermittent contact regime, which again proves our hypothesis that sixth harmonic is more sensitive to the stiffness. As the phase image shows, PS is in a full repulsive regime while PMMA is in an intermittent regime, the effect of Young's modulus is dominant in a repulsive regime that leads to the increase of the sixth harmonic of PS compared to PMMA. In this scenario, the impact of dissipation, is more pronounced for PS, leading to an increase of phase contrast as well. This can be easily seen by the high positive value of SSIM between the sixth harmonic and sin(∅) that shows the higher similarity between the two images, as presented in Figure 4b‐v.

**Figure 5:**
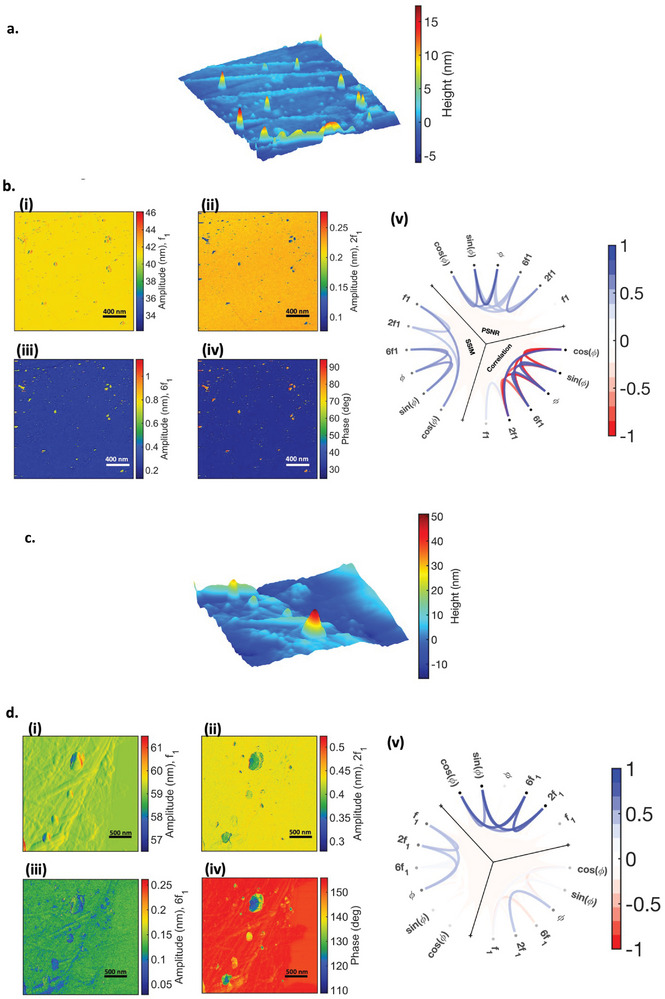
Nano Gold on HOPG sample. a) topography scanned over 2µ × 2µ section, b. i‐iv showing amplitude, 2nd and 6th harmonics, phase, and schemaball representing the correlation, PSNR, and SSIM between the WT‐derived images, **GO‐HOPG sample** c) topography scanned over 2.5µ × 2.5µ section, d) i‐iv showing amplitude, 2nd and 6th harmonics, phase, and schemaball representing the correlation, PSNR, and SSIM between the WT‐derived images.

In our investigation of two hard materials with different Hamaker constants, we examined nanogold particles deposited on the HOPG surface (**Figure** **5**). Our results reveal a contrast reversal between the second and sixth harmonics in the cantilever response – see Figure 5b‐ii,iii, as also presented by negative correlation in Figure 5b‐v. As observed from the phase image, the interaction is in intermittent contact, with HOPG in repulsive mode while gold is in an intermittent regime combined with both attractive and repulsive forces contributions. Here, HOPG, which is softer than gold, generates higher second harmonics, while the sixth harmonic of gold surpasses that of HOPG.

**Figure 6:**
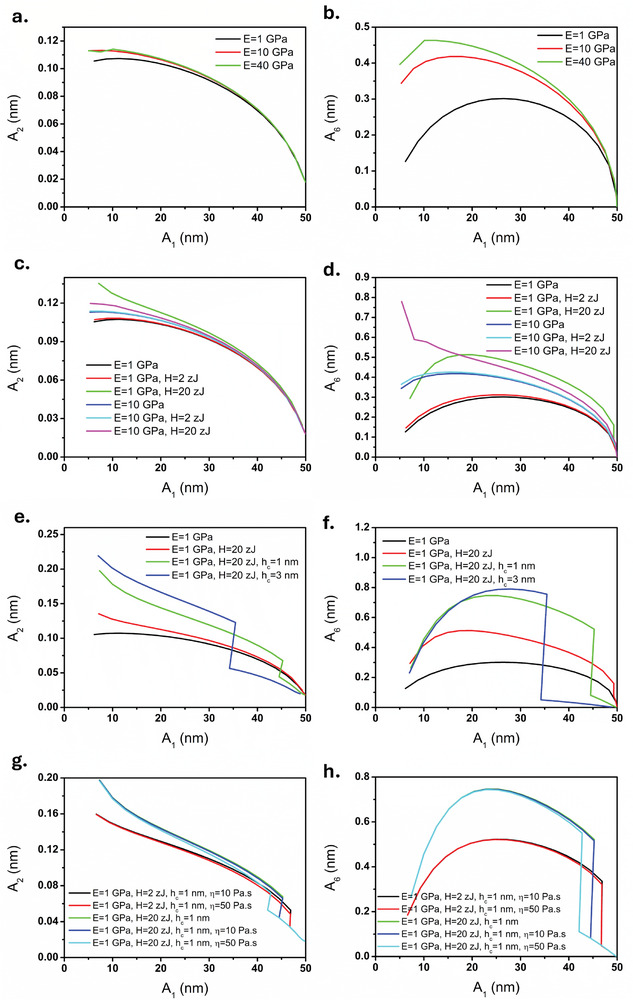
Simulation results of second and sixth harmonics at different conditions, a) second harmonic, b) sixth harmonic for different Young's modulus. c) second harmonic and d) sixth harmonic of different Young's modulus and Hamaker constant. e) second harmonic and f) sixth harmonic of the same Young's modulus for varying capillary force. g) second harmonic and h) sixth harmonic of the same Young's modulus, varying Hamaker constant and viscosity.

This phenomenon can be attributed to the interplay between the Hamaker constant (or adhesion) and Young's modulus, as well as indentation, consistent with the observations in the case of PS‐LDPE and simulations presented in subsequent figures. Furthermore, we examined our hypothesis by considering GO deposited on HOPG while we measured both samples in the intermittent regime (Figure 5c,d). As in this interaction regime, there is an interplay between both attractive and repulsive forces and despite higher Young's modulus of GO, in both the second and sixth harmonics, HOPG generated higher harmonics. This result is consistent with our simulation (**Figure** **6**c,d). Based on the traditional assumption, it is expected GO produces more harmonics than HOPG while these results clearly demonstrate the significant effect of the attractive force in generating the harmonics in intermittent contact regime.

To analyze and summarize our measured data, we performed a cascade‐PCA analysis. In the first layer of PCA, the input includes AFM‐measured parameters such as the amplitude of the main frequency, second and sixth harmonics, and phase. The output of the first layer is then used as the input for the second layer to establish the relationship between changes in measured AFM observables and material properties, including height, Young's modulus, Hamaker constant, and viscosity. The results are illustrated in Figure S4 (Supporting Information). Our c‐PCA results also are consistent with our proposed hypothesis (more information is given in Supporting Information). It is important to note that PCA is based on linear relationships between parameters, and in AFM measurements, the relation between observables and material properties is non‐linear. Therefore, our unsupervised learning results provide a qualitative explanation and analysis.

For a comprehensive understanding of the interplay between material properties and harmonics response, we conducted several simulations. We began by considering only Young's modulus and then incorporated the effects of Hamaker constant, capillary force, and viscosity into the material properties. The results are depicted in Figure 6.

In the case of the repulsive regime, we first examine the influence of Young's modulus alone (Figure 6a,b). As expected, an increase in Young's modulus leads to a rise in harmonic generation. This behavior is particularly evident in liquid environments, where such results are anticipated.^[^ ^53^ ^]^ Specifically, for both the second and sixth harmonics, raising the Young's modulus from 1 to 40 GPa increases the harmonic response. However, the sixth harmonic shows a higher sensitivity to changes in sample stiffness compared to the second harmonic. This observation is consistent with experimental results, such as those seen in the PS‐LDPE and PS‐PMMA systems (Figures 2 and 4), where the sixth harmonic demonstrates a greater sensitivity to variations in stiffness.

In the next phase of our simulation, we introduced the van der Waals (vdW) force, incorporating the Hamaker constant to represent attractive interactions between the vibrating tip and sample (Figure 6c,d). Here, we simulated six different scenarios by varying the Young's modulus (1 and 10 GPa) and adjusting the Hamaker constant across three levels: 0, 2, and 20 zJ. As the Hamaker constant increased, the expected trend shifted. For softer materials (1 GPa), the Hamaker constant had a more significant impact on harmonic generation, leading to increased contact between the cantilever and the surface and, consequently, higher harmonics compared to stiffer materials (10 GPa) similar to our experimental results of Figure 5c,d. Notably, at a certain setpoint, we observed contrast reversal in the sixth harmonic, which correlates with the experimental observations in Figures 2, 4, and 5b. This reversal is driven by the higher sensitivity of the sixth harmonic to adhesion forces at intermittent contact regime, particularly as indentation increases.

We then explored the effect of capillary forces on the harmonic response of the microcantilever by simulating interactions with capillary layers increasing from 1 nm to 3 nm in thickness. In this simulation, we maintained a Young's modulus of 1 GPa and a Hamaker constant of 20 zJ. We then introduced capillary forces to the interaction model, with two different capillary layer thicknesses. As shown in Figure 6e,f, the addition of attractive forces—by incorporating only the Hamaker constant—led to an increase in both the second and sixth harmonics. However, the inclusion of capillary forces significantly altered the dominance of the attractive regime, particularly before the tip indents the surface. Under the same high setpoints (for example >70% for 3 nm capillary layer and >90% for 1 nm capillary layer), the capillary force keeps the interaction in the attractive regime, reducing harmonic generation since there is no repulsive contact between the tip and the surface, meaning the stiffness contribution to harmonic generation is absent.

Furthermore, the presence of capillary forces induced a significant transition in harmonic responses, moving from the attractive regime to intermittent and repulsive regimes. This shift occurs due to the introduction of repulsive forces by reducing the setpoint, which, in conjunction with sample stiffness, Hamaker constant, and capillary forces, influences harmonic generation. After the tip contacts the surface, increasing the capillary force results in a rise in second harmonic generation, whereas for the sixth harmonic, a reversal of contrast occurs beyond a specific setpoint. This contrast reversal is consistent with our experimental findings (Figures 2, 4, and 5b). It is important to note that in experimental setups, it is impossible to isolate the effects of vdW and capillary forces; albeit phase images can confirm the presence of attractive forces. Thus, this simulation provides a complementary analysis, helping to distinguish and compare the contributions of vdW and capillary forces to harmonic generation and their roles in the observed anomalous contrast reversals.

Finally, we investigated the impact of viscosity on harmonic responses to correlate with our experimental observations (Figures 2 and 4). We modeled five cases, varying viscosity from 0 to 10 Pas, and then to 50 Pas (Figure 6g,h), while also accounting for the presence of a 1 nm capillary layer and two scenarios for the Hamaker constant (2 and 20 zJ). Our findings confirm that the presence of both capillary and vdW forces extends the interaction into the intermittent contact regime and increases both the second and sixth harmonics, aligning with experimental results, especially Figure 5b. However, viscosity introduces a reverse effect on harmonic response—an increase in viscosity leads to a slight reduction in harmonic amplitudes, which is consistent with the experimental results presented in Figures 2 and 4.

## 3 Conclusion

In this paper, we study the dynamics of vibrating solid‐solid interface interacting at the nanoscale through a comprehensive approach integrating experiments, simulations, and data analysis. Our investigation uncovers an interplay between vibrating solid‐solid interfaces, shedding light on nonlinear relationships governing the generation of harmonics within this regime. Our findings reveal a compelling nonlinear correlation between the generated harmonics and the interaction regime of the vibrating solid‐solid interface. Specifically, we elucidate that harmonics closer to the second eigenfrequency of the microcantilever exhibit heightened sensitivity to the stiffness of the sample under study. Furthermore, we identify the presence of two distinct repulsive and intermittent regimes during the scanning of the surface, leading to a notable contrast reversal between the observed harmonics. Of particular significance is our revelation of the overlooked impact of attractive forces, notably van der Waals forces, in the generation of harmonics. This aspect, traditionally neglected in the analysis of harmonics in microcantilever‐based AFM measurements, emerges as a significant contributor to the observed phenomena. Through our integrated approach, we provide novel insights into the complex dynamics governing solid‐solid interactions at the nanoscale, enriching our understanding of fundamental phenomena and paving the way for enhanced techniques in nanomechanical characterization.

## 4 Experimental Section

### AFM Simulation

To model the dynamics of the cantilever, a two degree of freedom model is chosen:^[^ ^45^, ^54^, ^55^ ^]^

(1) $$\frac{k_{i}}{\omega_{i}^{2}}{\ddot{q}}_{i}+\frac{k_{i}}{Q_{i}\omega_{i}}{\dot{q}}_{i}+k_{i}\;q_{i}=F_{di}\;\cos\left(\omega_{d}t\right)+F_{ts}\left(d,t\right)$$

where subscripts *i* = 1, and 2 denote to the first and second eigenmode respectively, *q_i_*, *k_i_*, *Q_i_* and ω_*i*_ are tip deflection, cantilever stiffness, quality factor, and resonance frequencies of the cantilever, respectively. *F_di_* is the drive amplitudes on the first and second modes (*F*_*d*2_ =   − 0.55*F*_*d*1_), and ω_*d*_ is the drive frequency usually chosen equal to the first natural frequency of the cantilever. *F_ts_* is the interaction force between the cantilever‐tip system and the sample surface and *d*  = *Z_c_*  − *q* is the minimum distance between the tip and sample. *Z_c_* is the average distance between the cantilever and sample and *q*  = *q*_1_  + χ*q*_2_ where χ  =  3.47 was measured in the simulation. This is due to the detection of the cantilever by the optical lever that measures the slope at the end of the cantilever.^[^ ^56^ ^]^ Note that by setting *q*_2_ (*t*) =  0 the classical point‐mass model commonly used to model dynamic AFM will be obtained.^[^ ^57^ ^]^ In our simulation, the interaction force consists of attractive van der Waals and capillary forces and repulsive DMT and Kelvin‐Voigt models:^[^ ^45^, ^51^, ^57^ ^]^

(2) $$F_{\mathrm{tsc}}=\left\{\begin{array}{c} -\frac{A_{h}R_{t}}{6d^{2}}-\frac{4\pi \gamma_{H_{2}O}R_{t}}{1+d/h_{c}}d\geq a_{0}\\ -\frac{A_{h}R_{t}}{6{a_{0}}^{2}}-\frac{4\pi \gamma_{H_{2}O}R_{t}}{1+d/h_{c}}+\frac{4E_{\mathrm{eff}}\sqrt{R_{t}}}{3}{\left(a_{0}-d\right)}^{\frac{3}{2}}d<a_{0} \end{array}\right.$$

(3) $$F_{tsnc}=\left\{\begin{array}{c} 0\;d\geq a_{0}\\ -\eta \sqrt{R_{t}\left(a_{0}-d\right)}\dot{d}\;d<a_{0} \end{array}\right.$$

where *A_h_* is Hamaker constant, *R_t_* is the tip radius, *h_c_* is water layer formed on the sample surface, $\gamma_{H_{2}O}$ is the surface energy of water, *d* is the distance between cantilever tip and surface, *E_eff_* is the effective Young's modulus, η is the viscosity and *a*_0_ is intermolecular distance. More information about modeling capillary force is given in Zitzler et. al.^[^ ^57^ ^]^

### AFM Experiments

Two‐composites polymer samples have been employed for the present work, one consisting of polystyrene (PS) and low‐density polyethylene (LDPE) (PS‐LDPE‐12M, Bruker corporation), and a second of PS and Poly (methyl methacrylate) (PMMA), produced by spin coating with a 25%/75% ratio. The experiments were performed in air using a commercial AFM system (D3100 Nanoscope III Digital Instruments, now Bruker) in amplitude modulation AFM (tapping mode) equipped with signal access module (SAMIII Digital Instruments) through which real‐time cantilever vertical deflection and piezo‐actuator drive signals were acquired via data acquisition board (NI USB‐6366, DAQ Device, National Instruments, Austin, TX, USA). Images of 4 µm scan size and aspect ratio 1 and 8 (128 × 64 and 128 × 16 pixels) were acquired at a scan rate of 2 Hz, with a silicon probe for soft tapping mode (FMV‐A Bruker, spring constant 3.24 N m^−1^ – as calculated by Sader method,^[^ ^58^ ^]^ resonance frequency 77.5 kHz). The cantilever was vibrated by the piezoactuator at a drive frequency of 77.4 kHz and target amplitudes free from interaction with the sample varying from 31 to 50 nm. After engagement with the surface, the amplitude setpoint was selected at 85%, 70%, and 50% of the one recorded at 100 nm lift. On data acquisition completion, the tip radius of curvature was estimated by the acquisition of a 1 µm × 1 µm image on a qualification sample (TipCheck sample, BudgetSensors.com) and processing by tip characterization procedure with SPIP software. Sensitivity was calibrated by performing amplitude curves on the qualification sample obtaining a conversion value of 33 nm V^−1^. The Au‐decorated HOPG and GO‐decorated HOPG samples were investigated with the Asylum Research Oxford Jupiter system by employing the tapping mode in the air. Imaging was conducted using an FMV‐A cantilever (Bruker), which had dimensions of 225 µm in length, 30 µm in width, and 2.75 µm in thickness. The spring constant was measured to be 1.7 N m^−1^.

### Feedback Error

In AM‐AFM the probe is excited close to its resonance, brought toward the surface until the amplitude of the oscillation is reduced to a setpoint value, corresponding to a definite tip‐sample interaction force. The topography was recorded by scanning the sample and keeping the cantilever's oscillation amplitude at the setpoint value by means of a feedback loop. In fact, a change in tip‐sample distance yields a change in interaction force and hence frequency, amplitude, and phase. By monitoring the value of the oscillation amplitude, the feedback loop detects changes in amplitude (hence in interaction force, hence in tip‐sample distance, hence in topography) and accordingly lifts or lowers the probe holder over the surface in order to keep it constant. The probe holder movement was recorded and reflects the topography of the sample. Inherently the action of the feedback occurs at a time interval after the topography changes, both because of its finite response and the delay in change of the cantilever's oscillation that happens within a timescale t_AM_ = 2Q/f_0_. Such delay is the cause of error in recording the topography, which can be detected by monitoring the amplitude, phase or frequency signals. For instance, an ascending (descending) feature decreases (increases) the tip‐sample distance and before this change is detected and corrected by the feedback, for a period of time the oscillation amplitude is smaller (greater) than the setpoint value. In the present experiments, inherent delay due to oscillation lag is estimated to be about t_AM_ = 5ms, corresponding to ≈3 pixels of the closed loop image.

### Sample Preparation

*Materials*: Tetrachloroauric(III) acid trihydrate (HAuCl4·3H2O), Sodium hydroxide (NaOH), and acetic acid of AR grade were obtained from Sigma Aldrich and used as received without further purification. Highly Oriented Pyrolytic Graphite (HOPG) with a mosaic spread value of 3.5°±1.5° was procured from µmasch (MikroMasch Europe, operated by NanoAndMore GmbH). Prior to experimentation, the HOPG surface was cleaved using Scotch Magic Tape (3M, USA). Ultrapure deionized (DI) water from a Millipore Milli‐Q system, with an electrical resistivity of 18 MΩ, was employed for preparing the aqueous solutions.

*Preparation of Au‐decorated HOPG*: The synthesis of gold nanoparticles from HAuCl_4_ utilized a citrate‐mediated thermal reduction method. Specifically, 1 milliliter of a 0.175 m aqueous citric acid solution was added to a solution of 0.52 mm HAuCl_4_ and stirred continuously. After 12 s, to adjust the pH of the solution, 1 M aqueous NaOH solution was introduced, followed by cooling. The resulting gold nano‐dispersion was collected and stored for further use. A 1 mM aqueous dispersion containing gold nanoparticles (with an average particle size of approximately40 nm) was prepared. Subsequently, 20 microliters of the as‐prepared nanodispersion were drop‐cast onto the freshly cleaved HOPG sample. The resulting assembly was air‐dried at room temperature, and the HOPG specimen decorated with gold (Au) was then subjected to AFM measurement.

*Preparation of GO‐decorated HOPG*: GO powder was bought from Sigma‐Aldrich (Merck, UK), and a water‐based dispersion of 0.1 mg mL^−1^ concentration was made using ultrapure DI water with a resistivity of 18 MΩ cm (Millipore Milli‐Q system, USA). Subsequently, the dispersion was drop‐cast onto a freshly cleaved HOPG surface and allowed to dry under ambient conditions.

*Preparation of PS‐PMMA*: PS and PMMA with molecular weights (Mw) of 290 000 and 350 000 amu, respectively (Sigma–Aldrich, UK), were used to create the necessary 25%PS/75%PMMA weight‐to‐weight ratio (referred to as PS‐PMMA thereafter). In order to make a 3% casting solution of this demixed system 0.75 g of PS was mixed with 1.50 g of PMMA and 97 g (63.7 mL) of chloroform (119.38 g mol^−1^, Sigma–Aldrich, UK) added. The solution was then placed on an orbital shaker in a Wheaton bottle for 24 h to ensure full dissolution and mixing. Silicon wafers (Inseto, UK) were placed in a beaker containing 99% isopropanol (IPA) (Sigma–Aldrich, UK) and placed in an ultrasonic bath (Ultrawave Ltd., Cardiff, UK) for 5 min to remove any surface contamination, before deionized water was used to rinse the surface. Substrates were then dried in an oven operating at 70 °C, until required. Clean, dry substrates were placed in the vacuum chuck of a SCS G3P‐12 spin coating device (PiKem, UK), and the PS‐PMMA solution dispensed onto the wafer until a consistent liquid layer formed. The spin program was then initiated with the substrate subjected to a spin program increasing up to 6000 RPM throughout a 3‐min cycle. PS‐PMMA substrates were then treated with acetic acid (Sigma–Aldrich, Irvine, UK) for 30 s to remove a portion of the PMMA uppermost layer and better expose the PS islands within the demixed thin film.

### Wavelet Transform

CWT can be obtained by calculating the convolution of sliding and dilating mother wavelet, *ψ*(*t*), and the signal, *x*(*t*):

(4) $$W\left(t,s\right)=\frac{1}{s}\int \psi *\left(\frac{u-t}{s}\right)du$$

In Equation (4), *s* is defined as the scale (representative of frequency bands) *t* is the time shift of the mother wavelet, and *ψ* ^∗^ represents the complex conjugate of the *ψ*, where the dilation and translation parameters of *ψ* vary continuously. CWT uses band‐pass filters localized in *ω_s_* to transform the signal into different scales. However, filterbanks associated with CWT for detecting the amplitude of higher harmonics seem to suffer from deficiencies that result in fluctuations and inaccurate amplitude calculation. MODWPT, uses wavelet basis functions to create a tree structure of filter banks, which performs the decomposition on both approximation and detail coefficients and accordingly results in homogeneous frequency bands. MODWPT can be a suitable means in estimating the time‐invariant sub‐signal of higher harmonics.^[^ ^59^ ^]^ Therefore, in this study, MODWPT was used to decompose the signal up to 7 levels that results in 128 frequency bands using “db45” as the mother wavelet.^[^ ^21^ ^]^ Further, the corresponding frequency bands to the first, second, and sixth harmonics were extracted and analyzed by CWT to obtain the amplitude of these harmonics. Consequently, the corresponding images were constructed from the results obtained from CWT.

### Data Analysis

Objective image quality metrics use explicit numerical criteria and prior information or references to represent the quality of the information in an image expressed as statistical parameters.^[^ ^60^ ^]^ Three objective image quality metrics were used to study the interplay between interaction forces and material properties of each sample.

1. **Peak Signal to Noise Ratio (PSNR)** is defined as the ratio between the maximum possible value of the image, *A_max_*, and the present noise, *MSE*, defined in Equation (5) PSNR shows higher values when the image shows better quality and contrast compared to the reference image.
(5) $$PSNR=10\;\;\log\left(\frac{A_{max}^{2}}{MSE}\right)$$

where MSE is reference measure that represents the error between an image and a reference image. Hence, MSE between image *A* and its estimated image of $\hat{A}$ with *X* ∗ *Y* dimension is defined as follows:

(6) $$MSE=\frac{1}{XY}\sum_{i=1}^{X}\sum_{j=1}^{Y}{\left(\hat{A}\left(i,j\right)-A\left(i,j\right)\right)}^{2}$$

and *A_max_* is the highest pixel value in the image. Small values of PSNR shows high numerical differences between the two images under consideration.

1. **Structural Similarity Index Measure (SSIM)** considers the image degradation as the change in the structural information of the image and is defined as follows:
(7) $$SSIM\left(A,\hat{A}\right)={\left[l\left(A,\hat{A}\right)\right]}^{\alpha }{\left[c\left(A,\hat{A}\right)\right]}^{\beta }{\left[s\left(A,\hat{A}\right)\right]}^{\gamma }$$

where *l, c, and s* denote the luminance, contrast, and the structural terms of the images. α > 0, β > 0, and γ > 0 are constant values that are adjusted based on relative importance of each component. For simplicity they are considered to be 1.^[^ ^61^ ^]^ SSIM is defined based on the human visual system (HVS) that is calculated as a combination of the closeness of luminance, contrast, and the correlation between two images.^[^ ^62^ ^]^

1. **Correlation** represents the degree of association between two images and is calculated using Equation (8), as follows:
(8) $$r=\frac{\sum_{i=1}^{X}\sum_{j=1}^{Y}\left(A\left(i,j\right)-mean\left(A\right)\right)\left(\hat{A}\left(i,j\right)-mean\left(\hat{A}\right)\right)}{\sum_{i=1}^{X}\sum_{j=1}^{Y}{\left(A\left(i,j\right)-mean\left(A\right)\right)}^{2}\;\sum_{i=1}^{X}\sum_{j=1}^{Y}{\left(\hat{A}\left(i,j\right)-mean\left(\hat{A}\right)\right)}^{2}}$$

### PCA Analysis

The goal of cascaded Principal Component Analysis (c‐PCA) was to find a pattern between the characteristics of materials and different signal measures of the AFM (f_1_, 2f_1_, 6f_1_, and phase). Here, c‐PCA involves doing PCA twice: first, between experimental parameters extracted from AFM and then between theoretical materials properties and eigenvalues of the first PCA, i.e., the PCA conducted on exclusive experimental parameters. This helps to understand the connection between non‐experimental measured parameters, like theoretical or non‐AFM measured values of η, E, H for different materials and the experimental data. This ensures that variability and non‐linearity between experimental and non‐experimental values are accounted for, else the non‐experimental value would be a fixed value in single PCA. This PCA was performed using the multiple variable analysis tool built within GraphPad Prism 10.2 software. First, individual PCA was performed on 11 parameters extracted from AFM analysis in different materials, i.e., PCA was performed on LDPE, PS, PMMA, HOPG, and Au with f_1_, 2f_1_, 3f_1_, 4f_1_, 5f_1_, 6f_1_, f_2_ phase, sin (phase), cos (phase), and height. After PCA, the principal components (PC) are chosen based on the Kaiser‐Guttman rule, which allows to consider principal components with eigenvalues >1. In this first PCA, (PCA of LDPE, PS, PMMA, HOPG, and Au), PC1, and PC2, which have eigenvalues >1, together accounting for at least 78% of the variance in the data, and therefore the value of eigenvector for f_1_, 2f_1_, 3f_1_, 4f_1_, 5f_1_, 6f_1_, f_2_ phase, sin (phase), cos (phase), and height of the PC1 and PC2 components were used for the second PCA that consisted 3 non‐experimental values of η, E, H for the comparison. The result of the second PCA generates new PCs that are linear combinations of the 8 input variables (f_1_, 2f_1_, 6f_1_ phase, height, η, E, H). The correlation between the f_1_, 2f_1_, 6f_1_, phases and the materials properties are plotted in Figure 5c.

## Conflict of Interest

The authors declare no conflict of interest.

## Supporting information



Supporting Information

## Data Availability

The data that support the findings of this study are available from the corresponding author upon reasonable request.
