# Supplementary material for: Incongruous Harmonics of Vibrating Solid‐Solid Interface
Source: Small. 2024 Nov 17;21(10):2409410. doi: 10.1002/smll.202409410 (PMC11899492; doi:10.1002/smll.202409410)
Supplement: Supplementary file 1 — Supporting Information [file SMLL-21-2409410-s001.docx]

**Supplementary Information: Incongruous harmonics of vibrating solid-solid interface**

Pardis Biglarbeigi^1^, Alessio Morelli^2^, Gourav Bhattacharya^2^, Joanna Ward^2^, Dewar Finlay^2^, Nikhil Bhalla^2^, Amir Farokh Payam^2^

^1^ *Department of Pharmacology & Therapeutics, University of Liverpool, Whelan Building, Liverpool, L69 3GE, England, UK.*

^2^Nanotechnology and Integrated Bioengineering Centre, School of Engineering, Ulster University, BT15 1AP, Belfast, UK.

**Results and Discussion:**


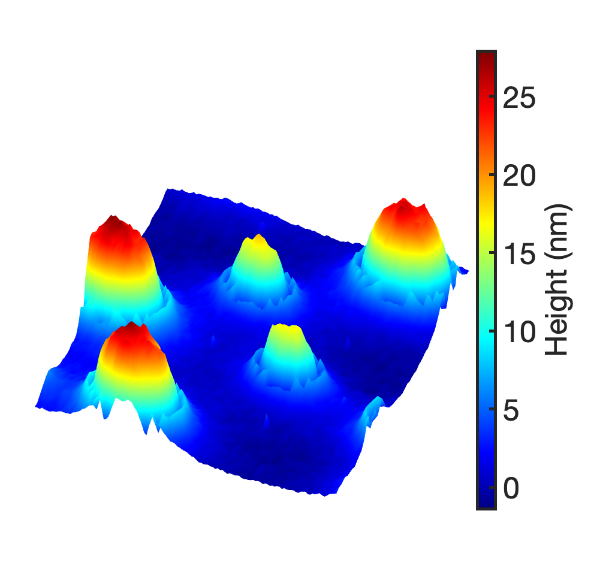


**a.**


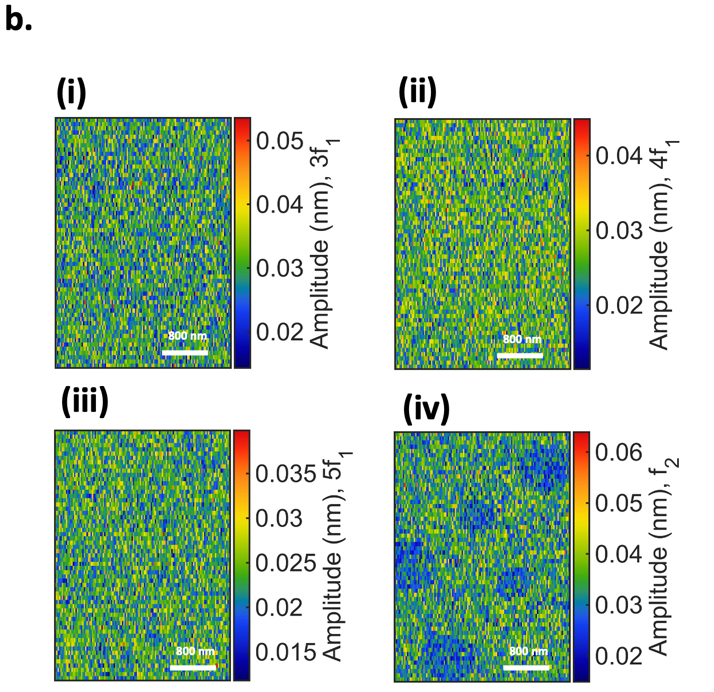

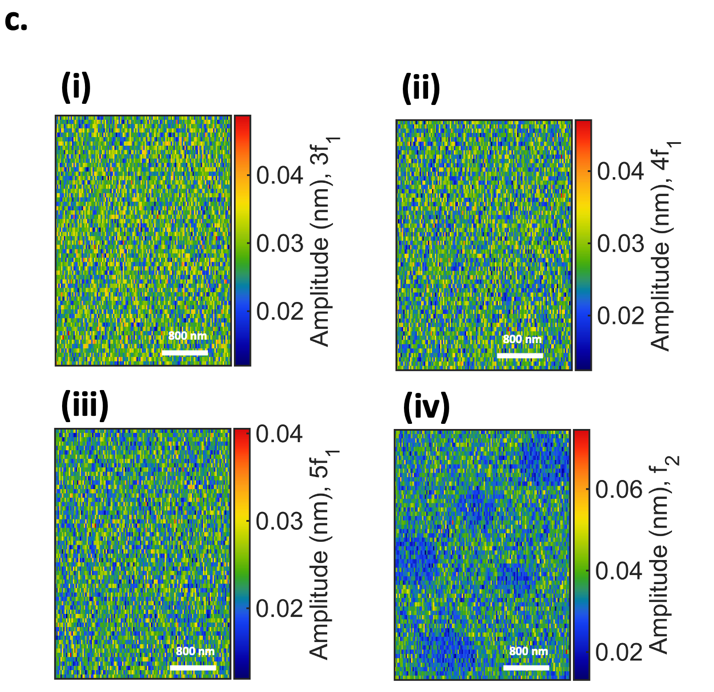


**Figure S1**. **PS-LDPE sample.** **a.** topography**. b.** 31 nm free amplitude at 50% set-point b.i.-b.iv. showing amplitude, 3^rd^ to 5^th^ harmonics, and second eigen harmonic **c.** 31 nm free amplitude at 85% set-point c.i.-c.iv. showing amplitude, 3^rd^ to 5^th^ harmonics, and second eigen harmonic.


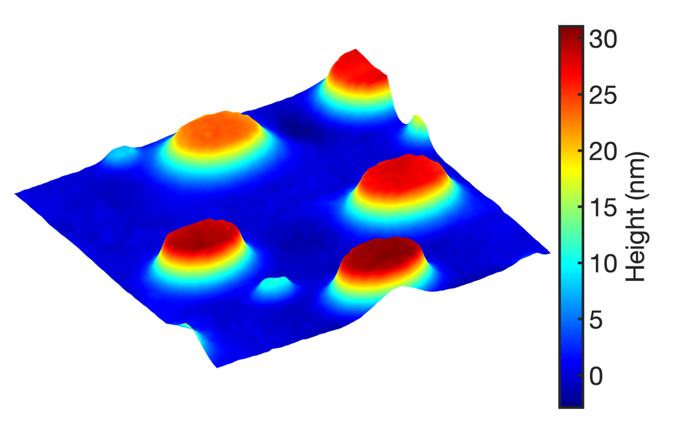


**a.**


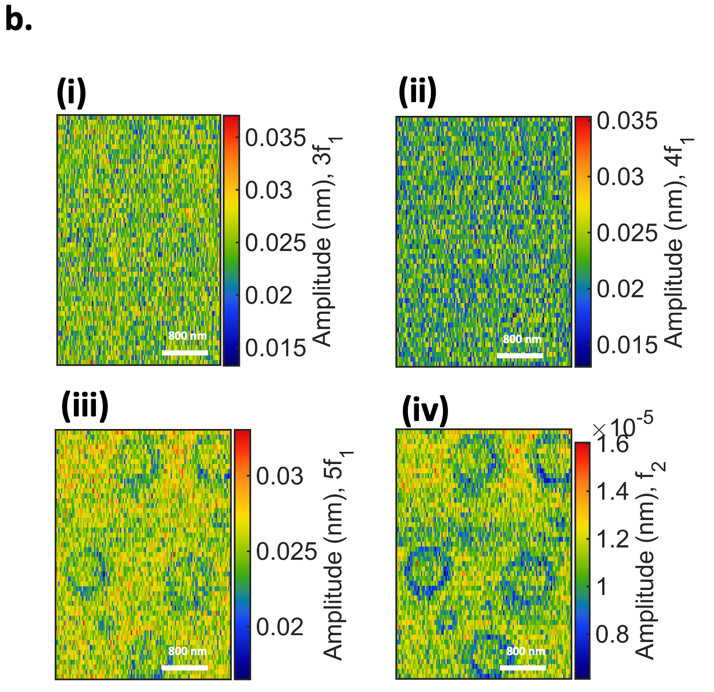

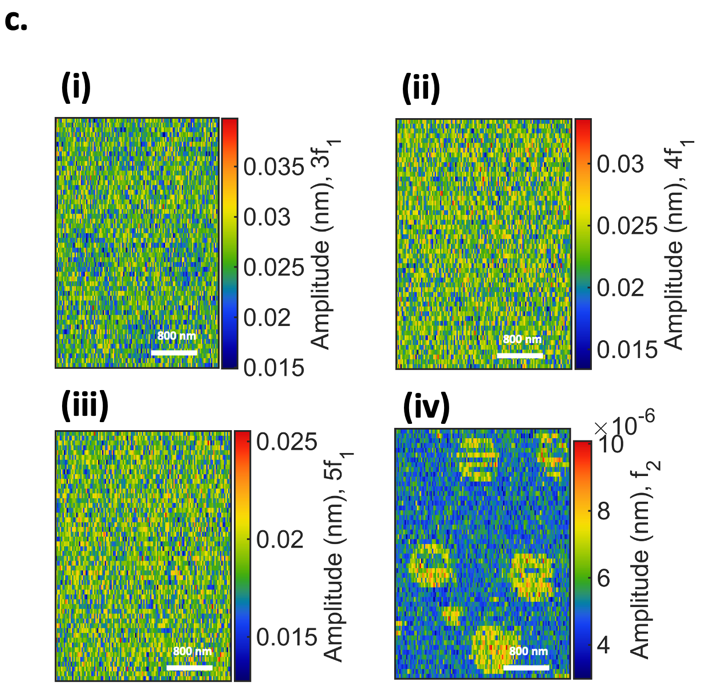


**Figure S2**. **PS-PMMA sample.** **a.** topography, **b.** 50 nm free amplitude at 50% set-point b.i.-b.iv. showing amplitude, 3^rd^ to 5^th^ harmonics, and second eigen harmonic **c.** 50 nm free amplitude at 85% set-point c.i.-c.iv. showing amplitude, 3^rd^ to 5^th^ harmonics, and second eigen harmonic.

**
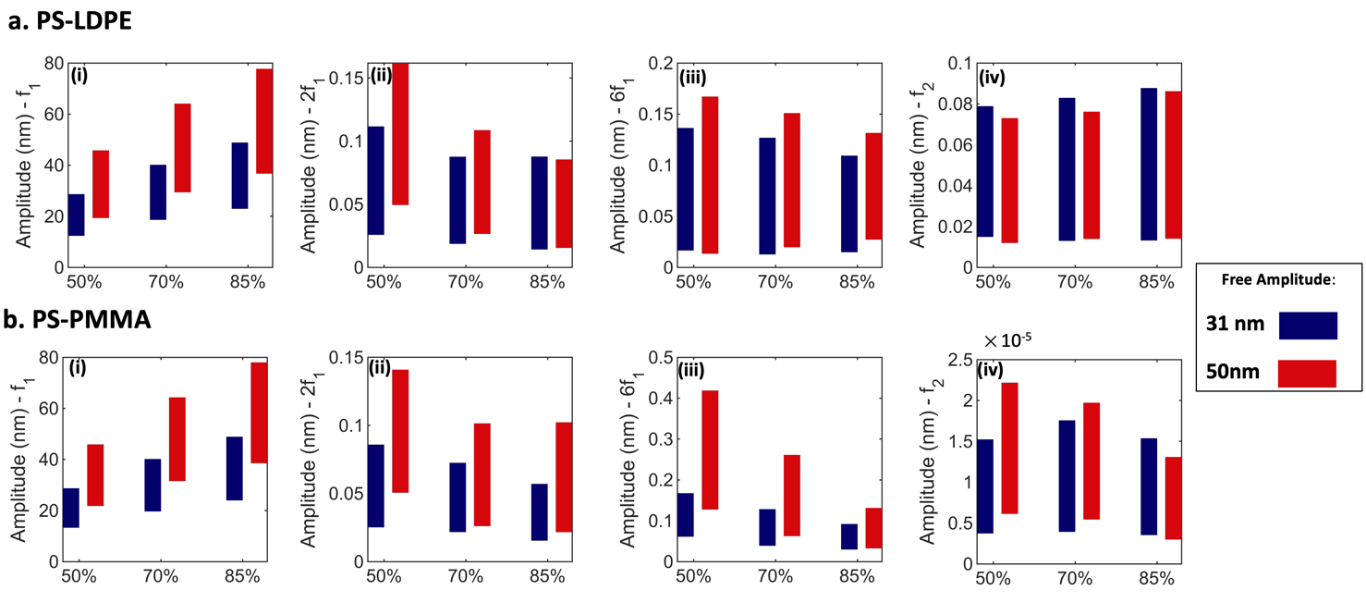
**

**Figure S3**. Range (min-max) of harmonics amplitudes for **a.** PS-LDPE and **b.** PS-PMMA samples.

**PCA Analysis Results**

As observed in our c-PCS analysis (Figure S4), height has a direct effect on the amplitude of the first resonance frequency and a reverse effect on phase, consistent with simulations and experiments. The phase is more influenced by viscosity, and the impact of Young’s modulus and Hamaker constant on the phase is low and indirect. This means that changes in height and Young’s modulus affect the harmonics, and according to previous findings^1,2^, as harmonics contribute to the phase change, the phase is affected.

Another interesting finding is the greater effect of the sixth harmonics on the phase, which is more influenced by Young’s modulus and Hamaker constant as it is close to the second eigenmode. This can be visualised in high positive values of correlation between phase and sixth harmonic, as well as high positive SSIM which shows a similarity between the two images, Figure 5.b.v. It is consistent with our measured data and analysis, the harmonics closer to the second eigenmode are more sensitive to changes in material properties, validated by our cascade-PCA results.

Furthermore, our results clearly indicate a contrast reversal between the second and sixth harmonics, as shown in Figures 5.b.ii and 5.b.iii. In the case that the tip experiences simultaneous attractive and repulsive interaction, depending on height and Young’s modulus, the response of the second and sixth harmonics to changes in Young’s modulus and Hamaker constant is reversed, while both exhibit the same trend regarding viscosity. These results directly address the contrast reversal observed in the second and sixth harmonics.

Regarding the amplitude of the main frequency, as seen, height has a direct effect as expected. Increasing Young’s modulus and Hamaker constant leads to a decrease in the main amplitude, and the effect of viscosity is negligible, consistent with previous reports^3^, simulations and experiments.


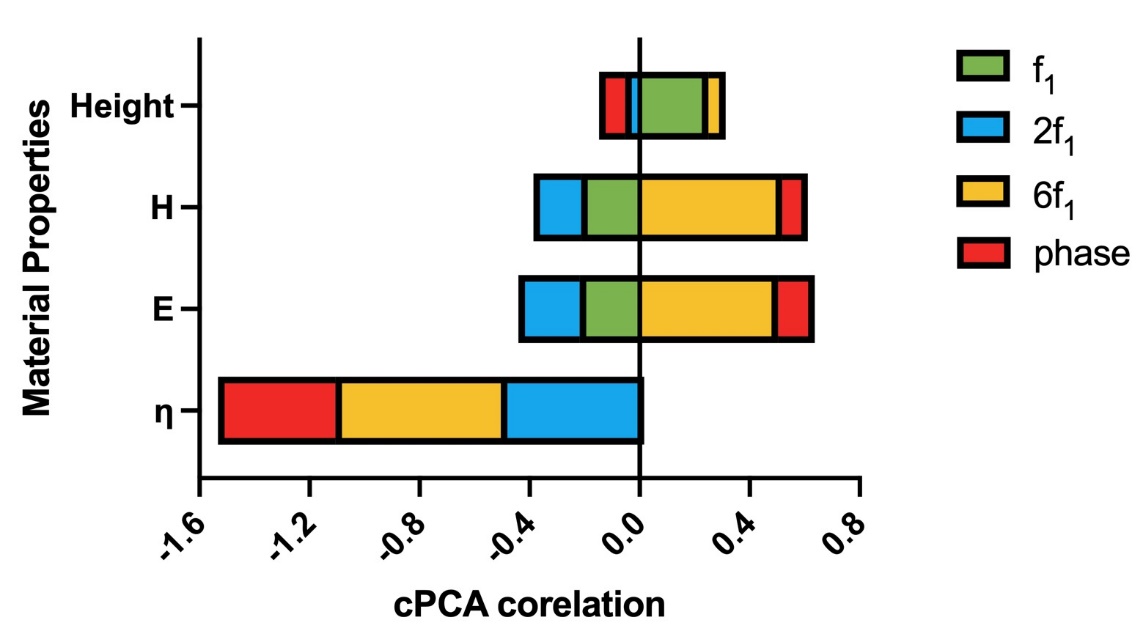


**Figure S4.** c-PCA analysis of our experimental results.

**References**

1. Sahin, O., Magonov, S., Su, C., Quate, C. F. & Solgaard, O. An atomic force microscope tip designed to measure time-varying nanomechanical forces. *Nat. Nanotechnol.* **2**, 507–514 (2007).

2. Payam, A. F., Ramos, J. R. & Garcia, R. Molecular and nanoscale compositional contrast of soft matter in liquid: Interplay between elastic and dissipative interactions. *ACS Nano* **6**, 4663–4670 (2012).

3. Farokh Payam, A. *et al.* Data acquisition and imaging using wavelet transform: a new path for high speed transient force microscopy. *Nanoscale Adv.* **3**, 383–398 (2021).
